# Continuous Monitoring of Vital Signs With Wearable Sensors During Daily Life Activities: Validation Study

**DOI:** 10.2196/30863

**Published:** 2022-01-07

**Authors:** Marjolein E Haveman, Mathilde C van Rossum, Roswita M E Vaseur, Claire van der Riet, Richte C L Schuurmann, Hermie J Hermens, Jean-Paul P M de Vries, Monique Tabak

**Affiliations:** 1 Department of Surgery University Medical Center Groningen University of Groningen Groningen Netherlands; 2 Department of Biomedical Signals and Systems University of Twente Enschede Netherlands; 3 Department of Cardiovascular and Respiratory Physiology University of Twente Enschede Netherlands; 4 eHealth group Roessingh Research and Development Enschede Netherlands

**Keywords:** wearable sensors, telemonitoring, continuous monitoring, vital signs, mHealth, wearable, biosensor, validity, accuracy

## Abstract

**Background:**

Continuous telemonitoring of vital signs in a clinical or home setting may lead to improved knowledge of patients’ baseline vital signs and earlier detection of patient deterioration, and it may also facilitate the migration of care toward home. Little is known about the performance of available wearable sensors, especially during daily life activities, although accurate technology is critical for clinical decision-making.

**Objective:**

The aim of this study is to assess the data availability, accuracy, and concurrent validity of vital sign data measured with wearable sensors in volunteers during various daily life activities in a simulated free-living environment.

**Methods:**

Volunteers were equipped with 4 wearable sensors (Everion placed on the left and right arms, VitalPatch, and Fitbit Charge 3) and 2 reference devices (Oxycon Mobile and iButton) to obtain continuous measurements of heart rate (HR), respiratory rate (RR), oxygen saturation (SpO_2_), and temperature. Participants performed standardized activities, including resting, walking, metronome breathing, chores, stationary cycling, and recovery afterward. Data availability was measured as the percentage of missing data. Accuracy was evaluated by the median absolute percentage error (MAPE) and concurrent validity using the Bland-Altman plot with mean difference and 95% limits of agreement (LoA).

**Results:**

A total of 20 volunteers (median age 64 years, range 20-74 years) were included. Data availability was high for all vital signs measured by VitalPatch and for HR and temperature measured by Everion. Data availability for HR was the lowest for Fitbit (4807/13,680, 35.14% missing data points). For SpO_2_ measured by Everion, median percentages of missing data of up to 100% were noted. The overall accuracy of HR was high for all wearable sensors, except during walking. For RR, an overall MAPE of 8.6% was noted for VitalPatch and that of 18.9% for Everion, with a higher MAPE noted during physical activity (up to 27.1%) for both sensors. The accuracy of temperature was high for VitalPatch (MAPE up to 1.7%), and it decreased for Everion (MAPE from 6.3% to 9%). Bland-Altman analyses showed small mean differences of VitalPatch for HR (0.1 beats/min [bpm]), RR (−0.1 breaths/min), and temperature (0.5 °C). Everion and Fitbit underestimated HR up to 5.3 (LoA of −39.0 to 28.3) bpm and 11.4 (LoA of −53.8 to 30.9) bpm, respectively. Everion had a small mean difference with large LoA (−10.8 to 10.4 breaths/min) for RR, underestimated SpO_2_ (>1%), and overestimated temperature up to 2.9 °C.

**Conclusions:**

Data availability, accuracy, and concurrent validity of the studied wearable sensors varied and differed according to activity. In this study, the accuracy of all sensors decreased with physical activity. Of the tested sensors, VitalPatch was found to be the most accurate and valid for vital signs monitoring.

## Introduction

### Background

Continuous telemonitoring of vital signs in daily life may lead to earlier detection of patient deterioration [1-3] and facilitate the migration of care toward home. In chronic diseases, telemonitoring is associated with improved clinical outcomes and cost-effectiveness of care [4,5]. It is expected that telemonitoring may also be of added value in other settings, such as the perioperative trajectory to monitor postoperative recovery in a ward or home setting. Preoperative monitoring at home may improve the knowledge of patients’ baseline vital signs. Especially since the COVID-19 pandemic, the demand for remote monitoring of vital signs has grown [6].

Several wearable sensors are available for telemonitoring of patients both in hospital and at home [1,7], which mainly differ in the location of placement, being reusable or disposable, battery life, and data transmission. According to legislation, sensors must be certified as a medical device and be safe and beneficial in their intended use. However, wearable sensors should be accurate and reliable as well before implementation in health care [1]. Accurate technology for telemonitoring is essential when used for clinical decision-making, although little is known about the accuracy and reliability of current generation wearable sensors, especially during daily life activities. Wearable sensors for continuous monitoring of vital signs are often evaluated in the in-patient setting [7] using patches (ie, Sensium Vitals, Sensium), mattress sensors (ie, EarlySense, EarlySense Inc), or more extensive sensors worn on the arm (ie, Radius-7, Masimo) [8]. Results from in-patient settings cannot directly be translated to the home environment when performing daily activities with less supervision, and research using wearable sensors for vital sign monitoring at home is lacking.

### Objectives

Information about the performance of wearable sensors in daily life is scarce and should be available before using these sensors for clinical decision-making. The aim of this study is to assess the data availability, accuracy, and concurrent validity of vital signs measured with currently available wearable sensors during daily life activities in a simulated living environment. We selected 3 types of recently available wearable sensors: arm-worn, chest-worn, and wrist-worn. This study investigates the technical performance of wearable sensors during daily life activities in volunteers to gain insight into their potential for telemonitoring.

## Methods

### Design

For this prospective observational validation study, experiments were performed at the eHealth House of the University of Twente, a simulated living environment (furnished apartment) used for research purposes [9]. The protocol was approved by the ethical committee of the University Medical Center Groningen and was executed according to the Declaration of Helsinki. Written consent was received from all participants for study participation and data use.

### Participants

Volunteers aged >18 years were included, with at least half of the participants aged >60 years, to reflect a general patient population. Interested volunteers were contacted by one of the researchers (RV) to assess their eligibility for study participation. The exclusion criteria were having a medical condition uncontrolled with medication that interferes with the execution of the protocol (ie, cardiovascular diseases, neuromuscular diseases, immobility, or cognitive disorders), pacemaker, or plaster allergy. Because of the lack of preliminary data for power calculation, a sample size of 20 was chosen on the basis of previous experience in quite similar validation studies for wearable devices associated with vital sign monitoring in volunteers [10-13].

### Devices

**Figure 1:**
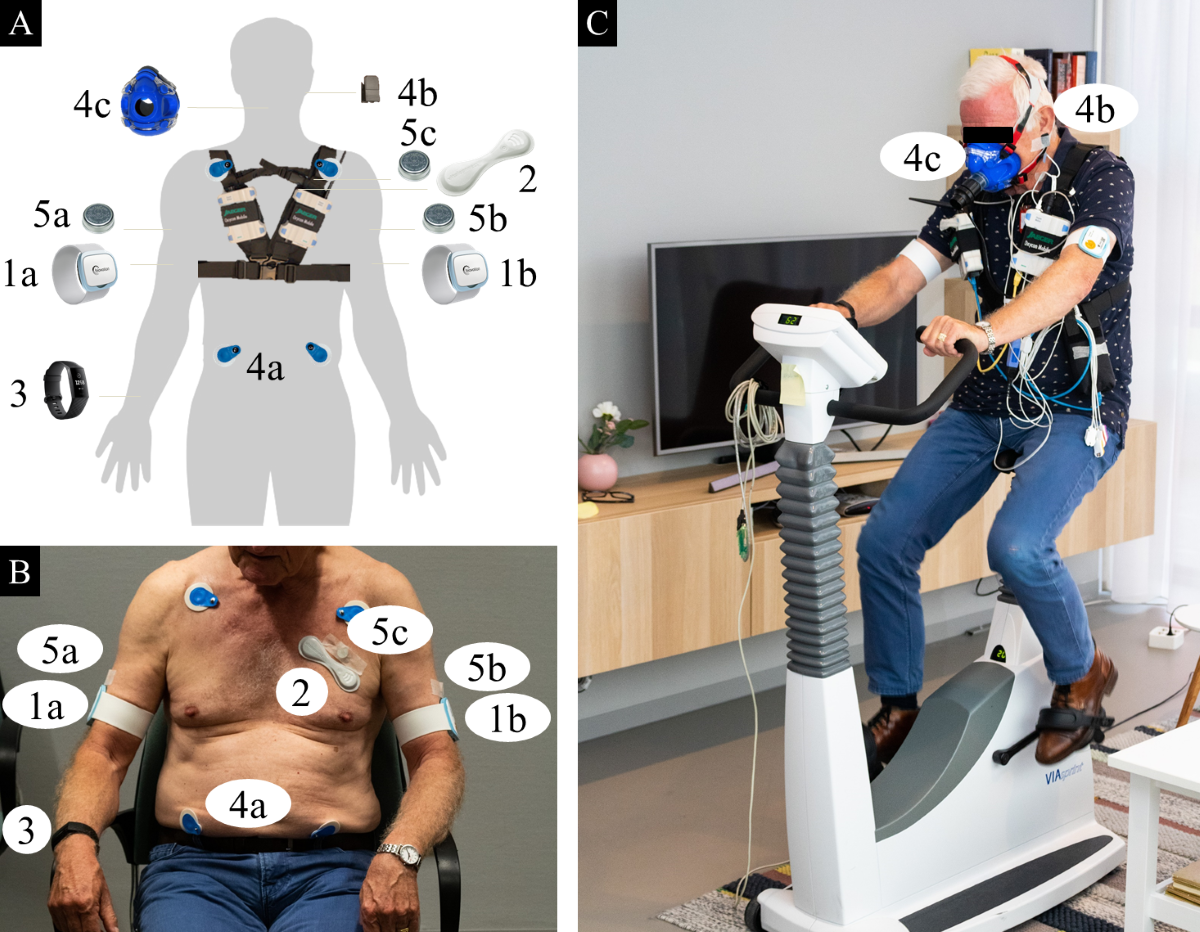
(A) Schematic overview of wearable sensors and reference devices and their placement on the participant’s body (B) during application and (C) during the experiment. Details: (1) Everion placed on the (a) right arm and (b) left arm; (2) VitalPatch; (3) Fitbit Charge 3; (4) Oxycon Mobile (a) 4 electrocardiography electrodes, (b) oxygen saturation probe, and (c) face mask; (5) iButton placed on the (a) right arm, (b) left arm, and (c) chest.

A total of 3 wearable sensors of interest for continuous and noninvasive measurement of vital signs were used: Everion (Biovotion AG), VitalPatch (MediBioSense), and Fitbit Charge 3 (Fitbit Inc). The VitalPatch is intended for the collection of physiological data in a health care setting, whereas Everion and Fitbit are intended to monitor fitness and general wellness only. These sensors differ in measurement location and techniques and have the potential to be used in clinical settings. All the used sensors are depicted in Figure 1.

Everion is a Conformity European (CE) class 2a–certified sensor worn on the upper arm that measures heart rate (HR), respiratory rate (RR), and blood oxygen saturation (SpO_2_) by photoplethysmography (PPG) and skin temperature using a negative temperature coefficient thermistor. The vital signs were stored every 10 seconds. VitalPatch is a CE class 2a–certified and Food and Drug Administration 510(k)–cleared disposable patch worn on the chest to measure HR and RR by electrocardiography (ECG) and temperature by a thermistor with a sample storage frequency of once per 4 seconds. The Fitbit Charge 3 is a commercially available activity tracker worn at the wrist and measures HR using PPG with a sample storage frequency of once per second during exercise and once per 5 seconds at all other times [14].

A total of 2 devices were used as gold standard reference devices. Oxycon Mobile (CareFusion Germany 234 GmbH) is a portable metabolic measurement system certified as a CE class 2a medical product and has been used as the gold standard in several studies [15,16]. Oxycon Mobile used ECG and expired volume measurements to monitor HR and RR, respectively. Volume measurement is a reliable method for RR calculation compared with other measurement principles that derive RR from impedance, ECG, or waveform modulation, such as in other wearable devices. In addition, SpO_2_ was measured using a PPG sensor that was positioned using an ear probe instead of a finger probe to enable free hand movement during the experiment. If ECG was missing, HR was determined from the SpO_2_ curve as reference. For all vital signs, a storage frequency of once per 5 seconds was used. The Thermochron iButton (Maxim Integrated), a validated wireless skin temperature logger [17], was used as a reference device for monitoring temperature with a sample storage frequency of once per 10 seconds and a resolution of 0.5 °C. The iButtons enabled wireless temperature measurements right above the relevant wearable sensors.

### Protocol

Before the start of the experiment, the protocol was explained and demographic data of participants were obtained and stored in Research Electronic Data Capture (REDCap; Vanderbilt University) version 10.0.23, including age, gender, BMI, occupation, physical activity lifestyle [18], and relevant medical history. The standardized protocol existed for 17 different tasks subsequently performed by participants with a total duration of 57 minutes. The detailed protocol, including task descriptions and durations, is provided in Multimedia Appendix 1. The task durations varied from 2 to 10 minutes and were performed in 6 activity clusters: resting, walking, metronome breathing, daily household activities (chores), stationary cycling on an exercise bike, and recovery. Transition periods were present between all tasks, which were not included in the data analysis. For more intensive tasks, a transition period of several minutes was included in the protocol for physiological stabilization between tasks. Resting included lying in several positions, sitting, and standing. Walking included walking at normal and slow speeds and stair climbing. Metronome breathing comprised breathing at 6, 15, 20, and 24 breaths per minute (brpm) and was guided by a metronome app. Chores were performed in the kitchen, where the participant was instructed to do various household tasks such as preparing food and cleaning. Cycling was performed on an ergometer with increasing load and rotation until a HR of at least 120 beats per minute (bpm) was reached. Thereafter, the participants recovered in an armchair or on a couch. During each experiment, 2 researchers were present, of whom 1 instructed the participant, and the other logged the start time of each task.

All sensors were synchronized with the computer time before the start of the experiment. During the experiment, vital signs were simultaneously recorded by the 4 wearable sensors and 2 reference devices. The placement of all the sensors is shown in Figure 1 and Table 1. A total of 2 Everion sensors were placed on the left and right arm, respectively, aiming to investigate the performance for different sensor placements. The data availability of real-time measurements was monitored regularly during the protocol, and technical issues were dissolved if needed.

**Table 1:** The 12 combinations of wearable sensors and reference devices to measure vital signs and their location on the participants’ body.

Vital sign and wearable sensor (location)		Reference device (location)
**Heart rate**		
	Everion (right upper arm)	Oxycon Mobile (4-lead ECG^a^/left ear lobe)
	Everion (left upper arm)	Oxycon Mobile (4-lead ECG/left ear lobe)
	VitalPatch (below left clavicular bone)	Oxycon Mobile (4-lead ECG/left ear lobe)
	Fitbit (right wrist)	Oxycon Mobile (4-lead ECG/left ear lobe)
**Respiratory rate**		
	Everion (right upper arm)	Oxycon Mobile (facemask)
	Everion (left upper arm)	Oxycon Mobile (facemask)
	VitalPatch (below left clavicular bone)	Oxycon Mobile (facemask)
**Oxygen saturation**		
	Everion (right upper arm)	Oxycon Mobile (left ear lobe)
	Everion (left upper arm)	Oxycon Mobile (left ear lobe)
**Skin temperature**		
	Everion (right upper arm)	iButton (right upper arm)
	Everion (left upper arm)	iButton (left upper arm)
	VitalPatch (below left clavicular bone)	iButton (below left clavicular bone)

^a^ECG: electrocardiography.

### Data Collection and Analysis

Data from all devices were exported from separate databases and processed in MATLAB R2018b (MathWorks, Inc) and SPSS Statistics 23 (IBM Corp). The logged start time and predefined duration of the respective tasks were used to select the data-recording windows for each task. Subsequently, nearest-neighbor resampling was used to pair wearable sensor data with the nearest data of reference devices for the combinations of sensors, as shown in Table 1. As the lowest data storage frequency was once per 10 seconds (for Everion and iButton), the maximum time shift between data points of the wearable sensor and reference device was 5 seconds. Data analysis was performed for each activity cluster and over the complete experiment (for all tasks).

### Statistical Analysis

#### Data Availability

The data availability of each sensor was assessed by the percentage of missing data points out of the expected data points per activity cluster and over all tasks per vital sign. In addition, the number and duration of missing data periods (epochs), for example, where the time between subsequent data points exceeded the expected sample period, was assessed.

#### Vital Sign Agreement

Agreement in vital sign data between wearable sensors and reference devices was inspected visually over all tasks. The measured values and variability of each sensor were described using the median and median absolute deviation (MAD) calculated per minute for all sensors and all participants. The median and IQR of the median and MAD of all participants were calculated per activity cluster and over all tasks to compare the (differences in) measured values and variability between activities and sensors. Furthermore, the median absolute percentage error (MAPE) was calculated per minute per vital sign to evaluate the accuracy of each wearable sensor.

#### Concurrent Validity

Concurrent validity was assessed using the data samples in a preselected activity cluster, with the aim of obtaining a large range of physiological variation with the least variation in position or task to minimize movement artifacts. Accordingly, the concurrent validity of HR was obtained in the cycling cluster, RR in the breathing cluster, and SpO_2_ and temperature in the recovery cluster after cycling. As the VitalPatch and Everion had an averaging duration of 45 and 60 seconds, respectively, to compute RR, measurements during the first minute of each breathing activity were not considered in the validity analysis. Before data selection, data of reference devices during the selected activity clusters were visually analyzed per participant to exclude physiologically implausible reference data by 2 researchers (MEH and MCVR). If needed, periods with unexpected scattering, variation, or drops were excluded from further analysis. Concurrent validity was assessed using Bland-Altman analyses to evaluate the mean differences (bias) and 95% limits of agreement (LoA). Bland-Altman analyses were corrected for repeated measurements, where the variance between measurement pairs was the sum of between- and within-subject variances [19,20]. The root mean square error (RMSE) was calculated to obtain insights into the amplitude of deviations. Bland-Altman plots, mean differences, LoA, and RMSEs were also assessed using median values per minute during the same predefined activity cluster. The results of both Bland-Altman analyses were compared to evaluate the influence of averaging on the concurrent validity of the wearable sensors.

## Results

### Overview

Between September 2020 and October 2020, 20 volunteers were included in the study. A total of 2 experiments were redone because of incomplete data from the reference devices because of recording failure. Data from 20 participants were analyzed, and the participant characteristics are shown in Table 2.

**Table 2:** Participant characteristics (N=20).

Characteristics			Values
Age (years), median (range)			64 (20-74)
**Age (years), n (%)**			
	20-40	4 (20)	
	40-60	4 (20)	
	60-70	7 (35)	
	70-80	5 (25)	
**Gender, n (%)**			
	Male	11 (55)	
	Female	9 (45)	
BMI (kg/m^2^), median (range)			23.4 (20.1-28.4)
**Physical activity lifestyle, n (%)**			
	Sedentary or light activity	10 (50)	
	Active or moderately active	10 (50)	
**Relevant medical history, n (%)**			
	No relevant medical history	14 (70)	
	Chronic obstructive pulmonary disease	2 (10)	
	Atrial fibrillation	1 (5)	
	Orthopedic surgery	3 (15)	

### Data Availability

**Figure 2:**
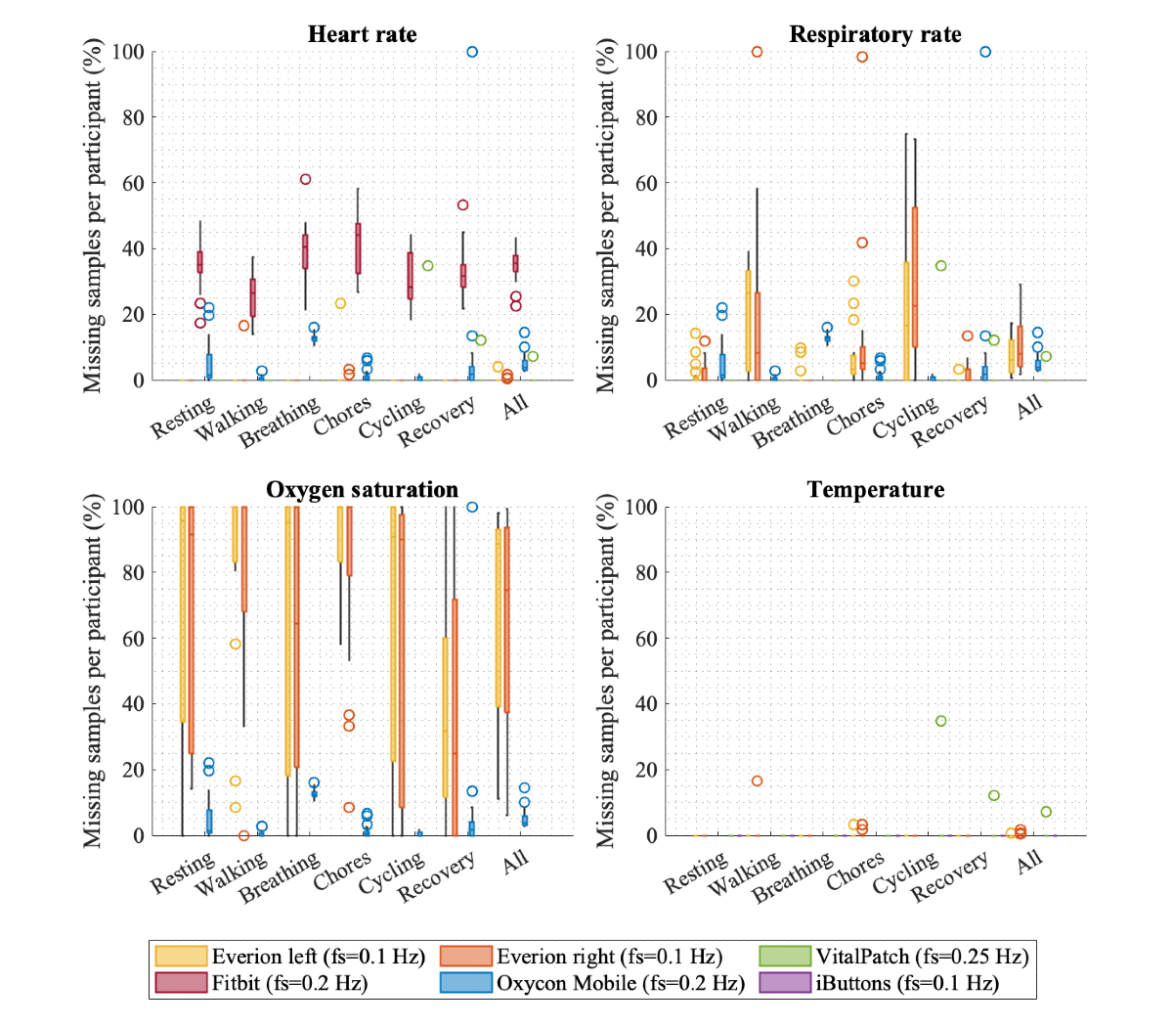
Boxplot (median, IQR, range, and outliers) of the percentages of missing samples per participant per activity cluster and over all tasks for each sensor based on its expected storage frequency per vital sign.

Percentages of missing samples are shown in boxplots per vital sign for the different activity clusters and all tasks in Figure 2. HR data measured by Everion was available 99.83% (13,657/13,680) of the time, where only 4% (1/23) of missing epochs was >30 seconds. VitalPatch measured HR 99.64% (17,039/17,100) of the time, with a maximum duration for missing epochs of 24 seconds. Data availability for VitalPatch was the same for all measured parameters; for example, data of all vital signs were available or none at all. Fitbit had the most missing data samples for HR based on the minimum-sample-storage frequency of once per 5 seconds; the median percentage of missing samples per participant was 35.7%. However, in 99.93% (2918/2920) of the missing epochs, the duration was ≤10 seconds.

For RR, Everion had the most available data during the breathing activity (2865/2880, 99.48%) and most missing data points during the more active clusters, walking and cycling, with median percentages of missing data of 8.3% to 26.4%. Of the missing RR epochs for both Everions, 51.4% (95/185) lasted >10 seconds, and 15.1% (28/185) lasted >1 minute up to 4 minutes.

SpO_2_ data by Everion were available 31.44% (4301/13,680) of the time (1960/6840, 28.66% at the left arm and 2341/6840, 34.23% at the right arm). Most SpO_2_ data of the Everions were recorded during recovery (753/1200, 62.75% of the time) and least during walking and chores activities, with a median percentage of missing data of 100%. Of all the missing SpO_2_ epochs for both Everions, 83% (93/112) lasted >10 seconds, and 16.1% (18/112) lasted >1 minute up to 9 minutes.

Temperature measurements of Everion were present 99.9% (13,669/13,680) of the time.

### Vital Sign Agreement

**Figure 3:**
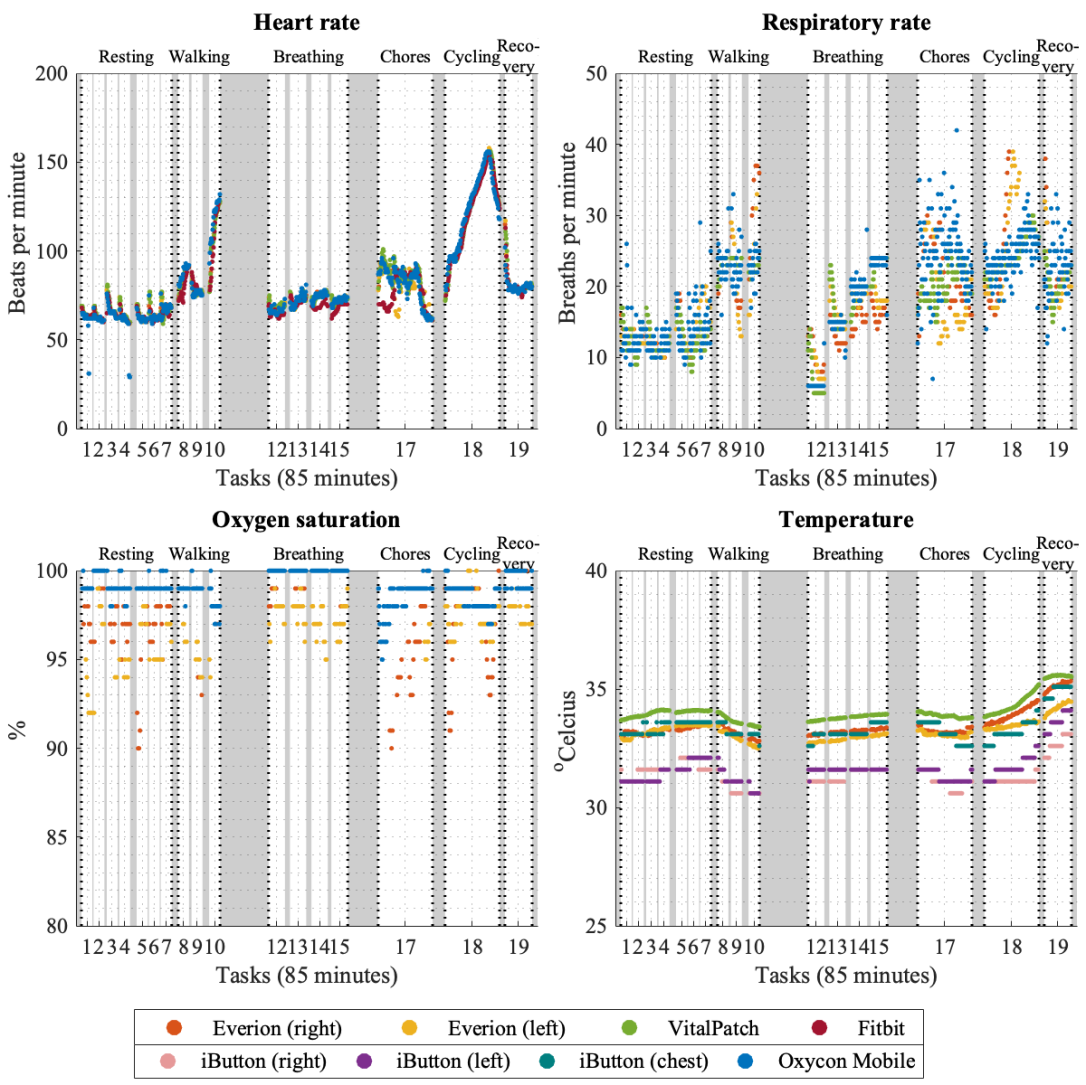
Measured data of all sensors for one study participant, classified by vital sign. White boxes represent the tasks, and the grey boxes the transition periods between tasks.

In most cases, wearable sensors showed similar trends compared with those of reference devices when measuring HR, RR, and temperature. Trends in vital signs during the complete experiment are shown in Figure 3 for 1 participant as an example. In half of the participants (10/20, 50%), an unexpected drop or low agreement in HR during cycling could be seen for Fitbit (9/10, 90%) and Everion (3/10, 30%), as illustrated in Multimedia Appendix 2, and also in the Oxycon Mobile (1/10, 10%).

The median values and MAD per minute for each vital sign and sensor over all tasks are shown in Table 3. Variability in terms of MAD per minute was generally low for all devices and vital signs.

**Table 3:** Median and median absolute deviation (MAD) per minute values of all participants for all sensors per vital sign over all tasks.

Parameters			Sensor, median (IQR)														
			Everion (right)		Everion (left)		VitalPatch		Fitbit		Oxycon Mobile		iButton (right)		iButton (left)		iButton (chest)
**Median**																	
	HR^a^ (bpm^b^)	78.5 (68-94)		78.5 (68-93)		79 (68-96)		76 (67-90)		80.5 (67.4-101.1)		N/A^c^		N/A		N/A	
	RR^d^ (brpm^e^)	17.5 (14-22)		18 (14.0-22.5)		18 (15-22)		N/A		19.5 (15.0-23.5)		N/A		N/A		N/A	
	SpO_2_^f^ (%)	98 (96-99)		98 (96.9-99)		N/A		N/A		99 (99-100)		N/A		N/A		N/A	
	Temperature (°C)	32.8 (31.9-33.8)		33 (32.2-33.9)		34 (33.4-34.6)		N/A		N/A		30.6 (29.6-31.6)		30.6 (29.6-31.6)		33.6 (33.1-34.1)	
**MAD**																	
	HR (bpm)	1 (0.5-2.0)		1 (0.5-2.0)		1 (1-2)		1 (1-2)		1.5 (1.0-3.5)		N/A		N/A		N/A	
	RR (brpm)	0.5 (0-1)		0.5 (0-1)		1 (0-1)		N/A		1 (0.5-2.0)		N/A		N/A		N/A	
	SpO_2_ (%)	0 (0-0.5)		0 (0-0.5)		N/A		N/A		0 (0-0)		N/A		N/A		N/A	
	Temperature (°C)	0 (0-0)		0 (0-0)		0 (0-0)		N/A		N/A		0 (0-0)		0 (0-0)		0 (0-0)	

^a^HR: heart rate.

^b^bpm: beats per minute.

^c^N/A: not applicable.

^d^RR: respiratory rate.

^e^brpm: breaths per minute.

^f^SpO_2:_ oxygen saturation.

The median MAPE of each wearable sensor as compared with the reference device per activity cluster per vital sign is shown in Table 4. For HR, all wearable sensors had an overall low median MAPE (2.3%-3.9%), with the highest MAPE for Fitbit. All sensors had the highest median MAPE during the walking cluster for HR (13.4%-23.4%). For RR, VitalPatch had the lowest median MAPE during the breathing and cycling cluster, whereas the Everion median MAPE was higher, especially during walking and cycling. The median MAPE of SpO_2_ measured by Everion was maximally 3.8% (during walking). The median MAPE for temperature of VitalPatch was very low (1%-1.7%). The lowest median MAPE for temperature of Everion was during the first activity cluster (resting: mean 6.3%) and the highest during the last cluster (recovery: mean 9%).

**Table 4:** Median absolute percentage error (MAPE) with IQR of all participants for all wearable sensors as compared with reference devices per vital sign during each activity cluster and overall tasks^a^.

Vital signs		Sensor (%), MAPE (IQR)					
		Everion (right)	Everion (left)	VitalPatch		Fitbit	
**HR^b^**							
	Resting	1.6 (0.7-4)	1.6 (0.7-4.4)	1.6 (0.7-4.8)		1.6 (0.6-4.1)	
	Walking	16 (2.8-33.3)	23.4 (3.1-35.2)	13.4 (3-32.6)		20.2 (8-34.4)	
	Breathing	2.1 (0.7-8.2)	2.2 (0.7-8.2)	2.7 (0.9-8.9)		3.2 (1.4-10.9)	
	Chores	2.1 (0.7-5.6)	2.6 (0.7-6.7)	1.7 (0.8-5.8)		6.2 (2.4-11.2)	
	Cycling	3 (1-6.2)	2.9 (0.8-6.7)	2.3 (1-4.9)		6.1 (2.4-14.8)	
	Recovery	1.1 (0.6-3.1)	1.1 (0.6-2.9)	1.3 (0-3.2)		1.6 (0.1-3.4)	
	All	2.3 (0.7-6.8)	2.3 (0.7-7.4)	2.3 (0.8-6.7)		3.9 (1.3-12)	
**RR^c^**							N/A^d^
	Resting	12.5 (6.1-21.9)	13.6 (6.5-22.6)	8.3 (4.8-14.3)			
	Walking	22.9 (10-46.7)	22.7 (9.7-41.7)	8.3 (4.2-15.7)			
	Breathing	20 (7.5-41.7)	20 (6.7-43.3)	6.7 (2.6-19.5)			
	Chores	19 (8.3-38.1)	22 (11.9-34.6)	15.9 (6.8-23.5)			
	Cycling	27.1 (13.2-42.8)	26.8 (13.2-42.6)	6.7 (3.6-11.9)			
	Recovery	12.5 (5.7-27.6)	14.6 (4.9-27.4)	7.7 (4.1-17.2)			
	All	17.5 (7.7-35.1)	18.9 (7.7-35)	8.6 (4.2-17.3)			
**SpO_2_^e^**					N/A		N/A
	Resting	2 (1-3.8)	2 (1-4)				
	Walking	3.8 (2.5-6)	3 (1.5-4.5)				
	Breathing	2 (1-2)	2 (1-3)				
	Chores	2.5 (1-3)	1 (1-2)				
	Cycling	1.5 (0.5-3)	1.3 (0.5-2)				
	Recovery	1 (0.5-2.5)	1 (0.5-2.1)				
	All	2 (1-3)	2 (1-3)				
**Temperature**							N/A
	Resting	5.9 (4.3-8)	6.7 (5.8-8.2)	1.1 (0.6-2)			
	Walking	7.8 (6.2-9.9)	8.8 (7.6-9.9)	1.2 (0.5-2.5)			
	Breathing	7.1 (5.9-9.5)	7.9 (6.2-9.6)	1 (0.5-1.7)			
	Chores	8.4 (6.5-10)	8.7 (6.9-10.4)	1.2 (0.7-2)			
	Cycling	8.2 (6.5-10.3)	9.1 (7-10.3)	1.7 (0.8-2.6)			
	Recovery	8.6 (6.7-10.7)	9.4 (7.6-11.4)	1.7 (0.7-3.1)			
	All	7.3 (5.9-9.6)	8.1 (6.5-9.9)	1.2 (0.6-2.3)			

^a^Median values per minute are used to calculate median absolute percentage error.

^b^HR: heart rate.

^c^RR: respiratory rate.

^d^N/A: not applicable.

^e^SpO_2_: oxygen saturation.

### Concurrent Validity

**Figure 4:**
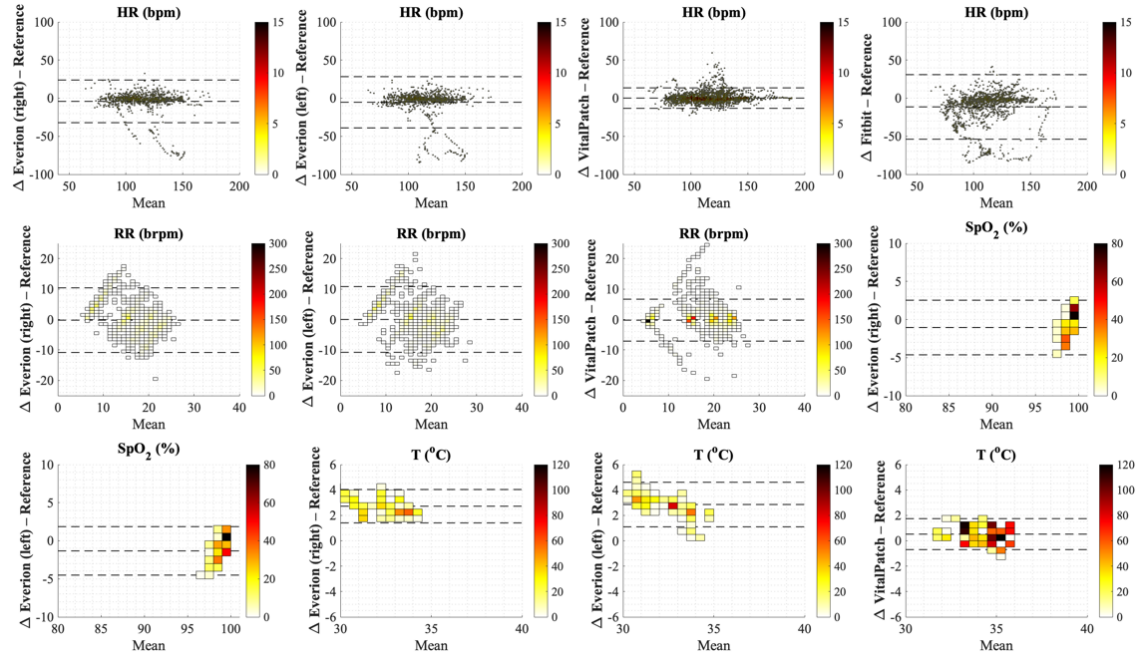
Bland-Altman plots for the 12 combinations of vital signs measured by the wearable sensors and reference devices for individual samples during the preselected activity cluster, where the x-axis represents the mean of and the y-axis the difference (Δ) between both sensors. Dotted lines represent the mean difference and limits of agreement for repeated measurements. bpm: beats per minute; brpm: breaths per minute; HR: heart rate; RR: respiratory rate; SpO_2_: oxygen saturation; T: temperature.

**Figure 5:**
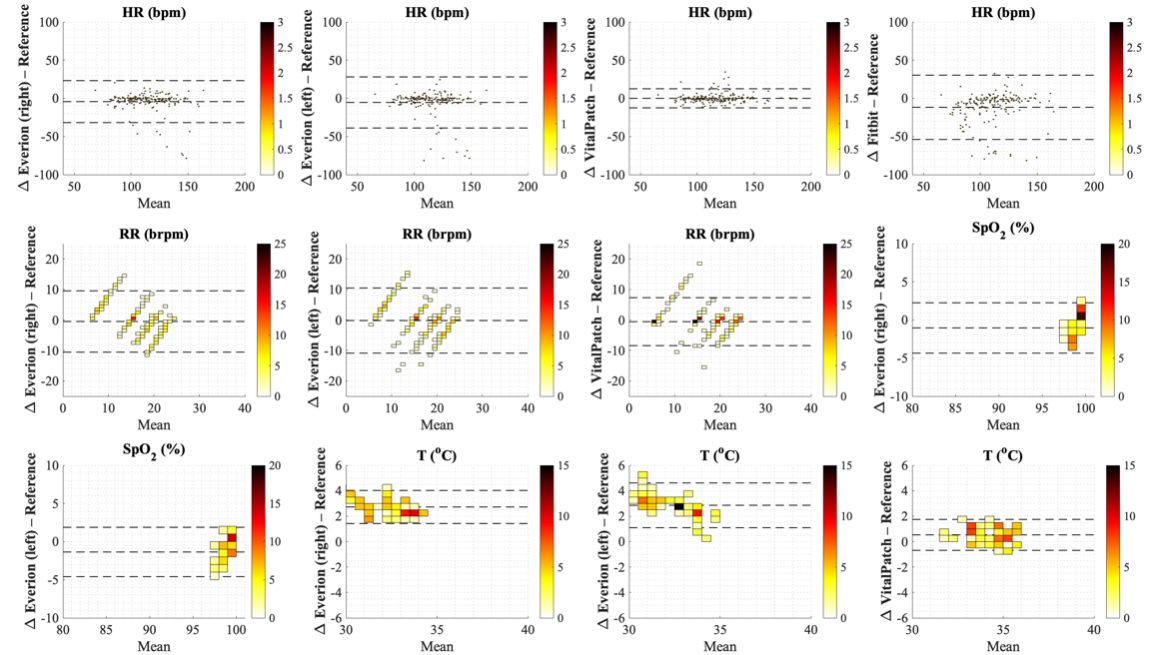
Bland-Altman plots for the 12 combinations of vital signs measured by the wearable sensors and reference devices of median data per minute during the preselected activity cluster, where the x-axis represents the mean of and the y-axis represents the difference (Δ) between both sensors. Dotted lines represent the bias and limits of agreement for the repeated measurements. bpm: beats per minute; brpm: breaths per minute; HR: heart rate; RR: respiratory rate; SpO_2_: oxygen saturation; T: temperature.

Figure 4 shows Bland-Altman plots for individual samples, whereas plots for median values per minute are shown in Figure 5. Table 5 shows mean differences and LoA from Bland-Atman analyses and RMSE per vital sign for the 2 methods for each wearable sensor compared with their reference devices.

**Table 5:** Mean differences and 95% limits of agreement (LoA) from Bland-Altman analysis for repeated measurements of each wearable sensor compared with its reference device per vital sign using both individual samples and median values per minute during the preselected activity cluster.

Vital signs			Everion (right)					Everion (left)					VitalPatch							Fitbit						
			Samples		Minute		Samples			Minute		Samples				Minute			Samples					Minute		
**HR^a^ (activity cluster: cycling)**																										
	Number of data pairs	1077		180		1076			180		2639				180			1490					179			
	Number of participants	18		18		18			18		18				18			18					18			
	Mean difference (LoA; bpm^b^)	−4.2 (−32.2 to 23.9)		−4.3 (−31.8 to 23.2)		−5.3 (−39 to 28.3)			−5.4 (−38.9 to 28.1)		0.1 (−13.3 to 13.5)				0 (−12.4 to 12.5)			−11.4 (−53.8 to 30.9)					−11.8 (−53.9 to 30.4)			
	RMSE^c^ (bpm)	14.7		14.5		17.7			17.6		6.8				6.3			24.1					24.1			
**RR^d^ (activity cluster: breathing)**																						N/A^e^				N/A
	Number of data pairs	2220		160		2102			160		3720				160											
	Number of participants	20		20		20			20		20				20											
	Mean difference (LoA; brpm^f^)	−0.4 (−10.6 to 9.8)		−0.5 (−9.1 to 8.0)		0.1 (−9.3 to 9.4)			−0.3 (−9.1 to 8.6)		−0.1 (−7.6 to 7.3)				−0.5 (−5 to 4.1)											
	RMSE (brpm)	5.5		5.1		5.6			5.4		3.5				4.0											
**SpO_2_^g^ (activity cluster: recovery)**														N/A			N/A				N/A				N/A	
	Number of data pairs	354		69		351			65																	
	Number of participants	17		17		17			17																	
	Mean difference (LoA; %)	−1.1 (−4.6 to 2.5)		−1 (−4.3 to 2.3)		−1.3 (−4.5 to 1.9)			−1.4 (−4.6 to 1.9)																	
	RMSE (%)	2.1		2.0		2.1			2.1																	
**Temperature (activity cluster: recovery)**																						N/A				N/A
	Number of data pairs	598		100		595			100		1478				100											
	Number of participants	20		20		20			20		20				20											
	Mean difference (LoA; °C)	2.7 (1.4 to 4.0)		2.7 (1.4 to 4.0)		2.9 (1.1 to 4.6)			2.9 (1.1 to 4.6)		0.5 (−0.7 to 1.7)				0.5 (−0.7 to 1.7)											
	RMSE (°C)	2.8		2.8		3.0			3.0		0.8				0.8											

^a^HR: heart rate.

^b^bpm: beats per minute.

^c^RMSE: root mean square error.

^d^RR: respiratory rate.

^e^N/A: not applicable.

^f^brpm: breaths per minute.

^g^SpO_2_: oxygen saturation.

For HR measured by VitalPatch, the mean difference was 0 bpm (LoA −13.3 to 13.5 bpm). Everion worn at the right or left arm underestimated HR with 4.2 bpm and 5.3 bpm (overall LoA −39.0 bpm to 28.3 bpm), and Fitbit underestimated HR with 11.4 bpm (LoA −53.8 bpm to 30.9 bpm).

Mean differences for RR were low with large LoA for both VitalPatch (LoA −7.6 brpm to 7.3 brpm) and Everion (LoA −10.6 brpm to 9.8 brpm). In addition, Figures 4 and 5 show higher differences for RR by Everion at the lowest breathing frequency (overestimation) and highest breathing frequency (underestimation).

SpO_2_ was underestimated, with mean differences of over 1% by Everion and LoA of −4.6% to 2.5%. For temperature, VitalPatch had a small overestimation of 0.5 °C. Everion overestimated temperature with a mean difference of 2.8 °C, with slightly higher differences at lower temperature and vice versa. The mean differences and LoA for median values per minute were similar to those for the individual samples.

## Discussion

### Principal Findings

Telemonitoring requires vital sign data from wearable sensors to be available, accurate, and valid when used for clinical decision-making, as well as during daily activities. Our results showed variable data availability and accuracy of vital signs measured for the evaluated wearable sensors during different daily life activities in a simulated free-living environment. VitalPatch is accurate and the least vulnerable to movement during daily activities. With regard to Everion, the mean difference, lower accuracy during physical activity, and limited data availability for RR and SpO_2_ must be considered when interpreting its measurements for diagnostic aims. Our results showed no relevant differences in performance between the left and right Everion because of sensor placement. Fitbit had a large mean difference and an activity-dependent storage frequency for HR.

Different results for the tested wearable sensors may be explained by differences in the underlying measurement technologies, processing algorithms, and sensor placement sites. Relevant findings and points of consideration will be discussed in the context of each sensor.

Our study showed low availability of Everion RR during the more active clusters and SpO_2_ data, which might be because of the placement site of the Everions. The upper arm is a nontraditional and uncommon site for measuring PPG signals, for which its accuracy has not yet been established [21,22]*.* Everion calculates an accuracy metric per vital sign, which prevents data with an accuracy <50% from being stored. This accuracy metric could be low when the measurement of vital signs is affected by movement, which is a general limitation of PPG signals [22,23]. On the other hand, the fact that Everion is PPG-based creates the ability to monitor multiple vital signs (HR, RR, and SpO_2_) with only 1 sensor [24]. There is an increasing demand for such devices, as patients are becoming multimorbid.

We reported an underestimation of HR by Everion. Only Barrios et al [13] evaluated HR measured by Everion in 6 healthy volunteers compared with ECG Holter measurements during different activities and found a mean difference for HR of −0.2 bpm (LoA of −6.3 bpm to 6.0 bpm) during cycling. These results imply better accuracy compared with those of our study, which could be related to the small number and young age of their participants. Finally, our study showed unexpected drops in HR by Everion during the rapid increase of HR while cycling without extensive arm movement, which is expected to be because of the algorithms of the sensors.

In our study, VitalPatch measured all vital signs with the highest accuracy and validity. No previous studies have been reported on the performance of VitalPatch. Only similar patches have been studied previously, including the Sensium Vitals patch (Sensium) [25] and HealthPatch (VitalConnect) [26].

For Fitbit, our study showed the lowest data availability of HR, which might be related to its irregular storage frequency. Although the sensor specification [14] stated that the sample storage frequency should be once per second to once per 5 seconds, depending on the level of activity, data were collected at much lower frequencies between once per 5 seconds and once per 15 seconds in our study.

Our results showed high errors and mean differences for Fitbit compared with the reference device. Earlier validation of the Fitbit Charge HR (Fitbit Inc) for HR showed higher accuracy during walking or running on a treadmill and lower accuracy during daily activities, with a MAPE of 8.4% and 10.1%, respectively [27]. A second validation study using Fitbit Charge HR showed an even higher underestimation of HR of 16 bpm during moderate-to-vigorous physical activity compared with Polar H6 HR monitor in 10 healthy participants during daily life activities [28].

In general, our results showed that the mean difference and LoA did not improve using median values per minute instead of individual data samples. This was unexpected, as using median values minimizes the influence of potential outliers. Breteler et al [26] found an improvement in the mean difference and LoA of HR and RR when applying a median filter per 15 minutes, although this might be more relevant for long-term measurements. In addition, averaging rigorously decreases the number of data points.

### Strengths and Limitations

A strength of this study is that we evaluated the sensor performance during daily life activities in a general population with mixed characteristics. In addition, the study was performed in a simulated home environment, which is as close as possible to the target setting while enabling well-controlled study measurements. Accordingly, the current results give more insight into the sensor performance as compared with typically performed validation protocols that only include young, healthy participants and measurements at rest.

A limitation is that we assessed the wearable sensor performance over a relatively short period. A second limitation is the limited translatability of our results to patients because of the measurement of vital signs in volunteers without pathophysiological abnormalities. Other limitations are related to the reference devices; we had to redo 1 volunteer because of the recording failure of Oxycon Mobile, and the resolution of the iButtons was set at 0.5 °C. This might have influenced the bias of Everion and VitalPatch in temperature. Although Oxycon Mobile has been used as the gold standard for portable monitoring of vital signs before [15,16], validation studies have so far focused on its measurement of metabolic capacity [29-33].

### Implications

Wearable sensors could assist in various areas of health care, such as detection of deviant values of vital signs to alarm health care professionals, trend analysis to monitor recovery or deterioration, and decision-making to operate or visit the hospital. Applications of vital sign telemonitoring are diverse, from trend monitoring to acute alarms, based on the clinical goal and which medical actions follow. The required accuracy of the sensor measurements depends on this. Sensor performance for patient monitoring still needs evaluation in specific patient groups, at home or in hospital, during longer periods, and on its diagnostic ability, which are the next steps toward clinical applicability. Patient acceptance and actual use (adherence) are important for clinical use [34]. Therefore, this should be the subject of future work. However, the potential of our tested wearable sensors for patient monitoring will be discussed in the context of the following technical factors to consider: (1) the vital signs to monitor, (2) a sensors’ accuracy and trending ability, (3) data storage frequency or filtering, and (4) confounding factors such as movement during daily activities.

First, the vital signs that need to be monitored depend on the aforementioned application. For example, for in-hospital monitoring, detection of cardiac events might require ECG monitoring [35], whereas for detection of postoperative deterioration, all vital signs used in the modified early warning score might be preferred [2], which are HR, RR, temperature, SpO_2_, and blood pressure. In many cases, it is still unknown which parameters to monitor at home and how to interpret long-term measurements obtained in a remote setting, as current common practice is often that a patient returns to or contacts the hospital in case of (increasing) symptoms without further monitoring [7,36]. The ability of the tested sensors to measure the available parameters is discussed per vital sign.

VitalPatch and Everion both monitor multiple vital signs, whereas VitalPatch can also monitor raw ECG. HR is the most commonly measured vital sign and is often measured accurately [7,8,13]. Owing to its large mean difference and unexpected drops during rapidly increasing HR, Fitbit is the least suitable for HR monitoring in patients.

Everion measurements for RR were less accurate <15 brpm or >20 brpm, according to our Bland-Altman analyses. However, these ranges are especially important for the detection of deterioration and predicting cardiac arrest [37,38]. Algorithms for ECG and PPG can use the same techniques to derive RR, such as amplitude and frequency modulation, although algorithms based on ECG perform better than those based on PPG [39]. Respiratory-synchronized variations are subtle, and proximity to the chest improves the measurement of RR (less susceptible to vasoconstriction) [40,41]. Therefore, VitalPatch may be preferred for monitoring RR.

SpO_2_ is less commonly measured [7,8,13]. Available wearable SpO_2_ sensors are generally commercially available fingertip sensors, and few meet the International Organization for Standardization 80601-2-61 accuracy standards [42]. Fingertip probes are not ideal for long-term monitoring of SpO_2_ at home, although this enables transmission mode PPG with higher perfusion compared with more convenient measurement sites that require reflection mode PPG [41,42]. The low variability in SpO_2_ levels of volunteers precludes insight into the accuracy of Everion for monitoring SpO_2_ levels in patients. However, because of its limited data availability and underestimation of SpO_2_, our results indicate that Everion is not suitable for (high-frequency) clinical monitoring of SpO_2_.

Most available wearable sensors measure skin temperature (including Everion and VitalPatch), whereas core temperature may be clinically more relevant because of its current use in clinical practice. Nevertheless, the clinical relevance of skin temperature monitoring should be evaluated in future research [2].

Second, it is important to define what performance and trending ability are acceptable for clinical use. Currently, no criteria are available for MAPE, mean differences, and LoA of wearable sensors. Although all wearable sensors in our study followed similar trends compared with those followed by the reference devices for HR, RR, and temperature, their trending ability and diagnostic ability to detect clinically relevant changes should be assessed during longer assessments in patients.

A challenge for validation studies for vital sign monitoring is choosing the right reference devices to use as gold standard devices [43]. We experienced that ECG cables and electrodes used for the HR reference measurements are susceptible to movement as well, as also described by Barrios et al [13] using ECG Holter. The Oxycon Mobile reference device enabled ambulatory expired volume analysis, which is the best available solution to monitor RR wireless and continuously. Accordingly, the RR validation results are expected to be more accurate as compared with those of clinical validation studies that use intermittent nurse assessments as reference, which is often poorly reported or inaccurate [1].

Third, optimal filtering strategies and data storage frequencies should be investigated. Fourth, further reduction of movement artifacts, for example, using information from the present accelerometer [42], is essential for optimizing measurements at sites that enable long-term monitoring, such as the upper arm.

### Conclusions

To use wearable sensors for clinical decision-making, information about their performance in daily life is needed. Of the tested sensors, VitalPatch was found to be the most accurate and valid for vital sign monitoring. For all sensors, movement during daily activities should be considered. Longer assessments of wearable sensors are needed to evaluate the technical performance and trending ability to work toward the clinical applicability of wearable sensors in patients.

## Appendix

Multimedia Appendix 1 Measurement protocol with the activity clusters, task descriptions and task durations and cumulative time of the 17 tasks included in the analysis.

Multimedia Appendix 2 Measured data of all sensors for one study participant, classified by vital sign. White boxes represent the tasks, and the grey boxes the transition periods between tasks.

## Abbreviations

bpm: beats per minute
brpm: breaths per minute
CE: Conformity European
ECG: electrocardiography
HR: heart rate
LoA: limits of agreement
MAD: median absolute deviation
MAPE: median absolute percentage error
PPG: photoplethysmography
REDCap: Research Electronic Data Capture
RMSE: root mean square error
RR: respiratory rate
SpO_2_: oxygen saturation
