# Supplementary material for: Continuous Monitoring of Vital Signs With Wearable Sensors During Daily Life Activities: Validation Study
Source: JMIR Form Res. 2022 Jan 7;6(1):e30863. doi: 10.2196/30863 (PMC8783291; doi:10.2196/30863)
Supplement: Multimedia Appendix 1 [file formative_v6i1e30863_app1.pdf]

Multimedia Appendix I. Measurement protocol with the activity clusters, task descriptions and task durations and cumulative time of the 17 tasks included in the analysis. Transition periods were present between all tasks, which were not included in data analysis. For more intensive tasks, a transition period of several minutes was included in the protocol for physiological stabilization between tasks.

**1 – Resting**				
**Task type**	**Task (#)**	**Description**	**Duration (min.)**	**Cumulative (min.)**
Rest	Lying (1-5)	The participant lies relaxed in supine position with his or her head on a pillow on the bed for two minutes. From there on the participant changes lying position to side, prone, side and lying on an inclined bed. Whereby, each lying position lasts 2 minutes.<br><br>*Supine – Side – Prone – Side – Lying on back on a inclined bed (45 deg.)*	10	10
Rest	Sitting (6)	After the first task the participant moves out of bed and takes a seat in the chair next to the bed. The participant now sits relaxed in the chair.	2	12
Rest	Standing (7)	The participant gets up from the chair and stands still and relaxed.	2	14

**2 – Walking**				
**Task type**	**Task (#)**	**Description**	**Duration (min.)**	**Cumulative (min)**
Active	Walking (8-9)	The participant now moves from the bedroom to the hallway outside the eHealth house. There he or she will walk back and forth in the hallway twice two minutes at different walking speed.<br><br>*Normal walking speed – Slow walking speed (strolling)*	4	18
Active	Stair climbing (10)	The participant moves to the closest stairs near the hallway and climbs up	2	20

Task type	Task (#)	Description	Duration (min.)	Cumulative (min)
		and down the stairs.<br><br>*Normal stair climbing speed*		
Transition	Sitting (-)	The participant goes back to the eHealth house and takes a seat in the chair at the dinner table. He or she will sit still and relaxed in the chair.	3	-
**3 – Metronome breathing**				
**Task type**	**Task (#)**	**Description**	**Duration (min.)**	**Cumulative (min)**
Metronome	Visually-guided metronome breathing (11-14)	The participant stays seated in the chair at the dinner table to do four metronome breathing exercises of three minutes each.<br><br>*6 breaths/min – 15 breaths/min – 20 breaths/min – 24 breaths/min*	12	32
Transition	Sitting (-)	After the last breathing exercise, the participant stays seated in the chair at the dinner table.	6	-
**4 – Daily life activities (chores)**				
**Task type**	**Task (#)**	**Description**	**Duration (min.)**	**Cumulative (min)**
Active	Chores (15)	Without a lot of instruction, the participant will have to do chores in the kitchen.<br><br>Setting the dinner table – making some tea – wiping countertops – washing hands – cleaning up dinner table – reading the newspaper/magazine	10	42
**5 – Cycling**				
**Task type**	**Task (#)**	**Description**	**Duration (min.)**	**Cumulative (min)**

Active	Cycling (16)	The participant moves to the living room where the 6 minutes submaximal test will take place on a cycle ergometer.<br><br>*Warming-up (2 minutes)*<br>Warm up on the ergometer cycle with low intensity.<br><br>*Test (6 minutes)*<br>The workload will be determined depending on the participants age, gender and physical condition. Intensity will be adjusted accordingly.<br><br>*Cooling down (2 minutes)*<br>Cool down on the ergometer cycle.	10	52
Rest	Recovery (17)	After cooling down in the cycle ergometer the participant will take a seat on the sofa in the living room, sitting still and relaxed.	5	**57**
