# Supplementary figures and images for: Continuous Monitoring of Vital Signs With Wearable Sensors During Daily Life Activities: Validation Study
Source: JMIR Form Res. 2022 Jan 7;6(1):e30863. doi: 10.2196/30863 (PMC8783291; doi:10.2196/30863)

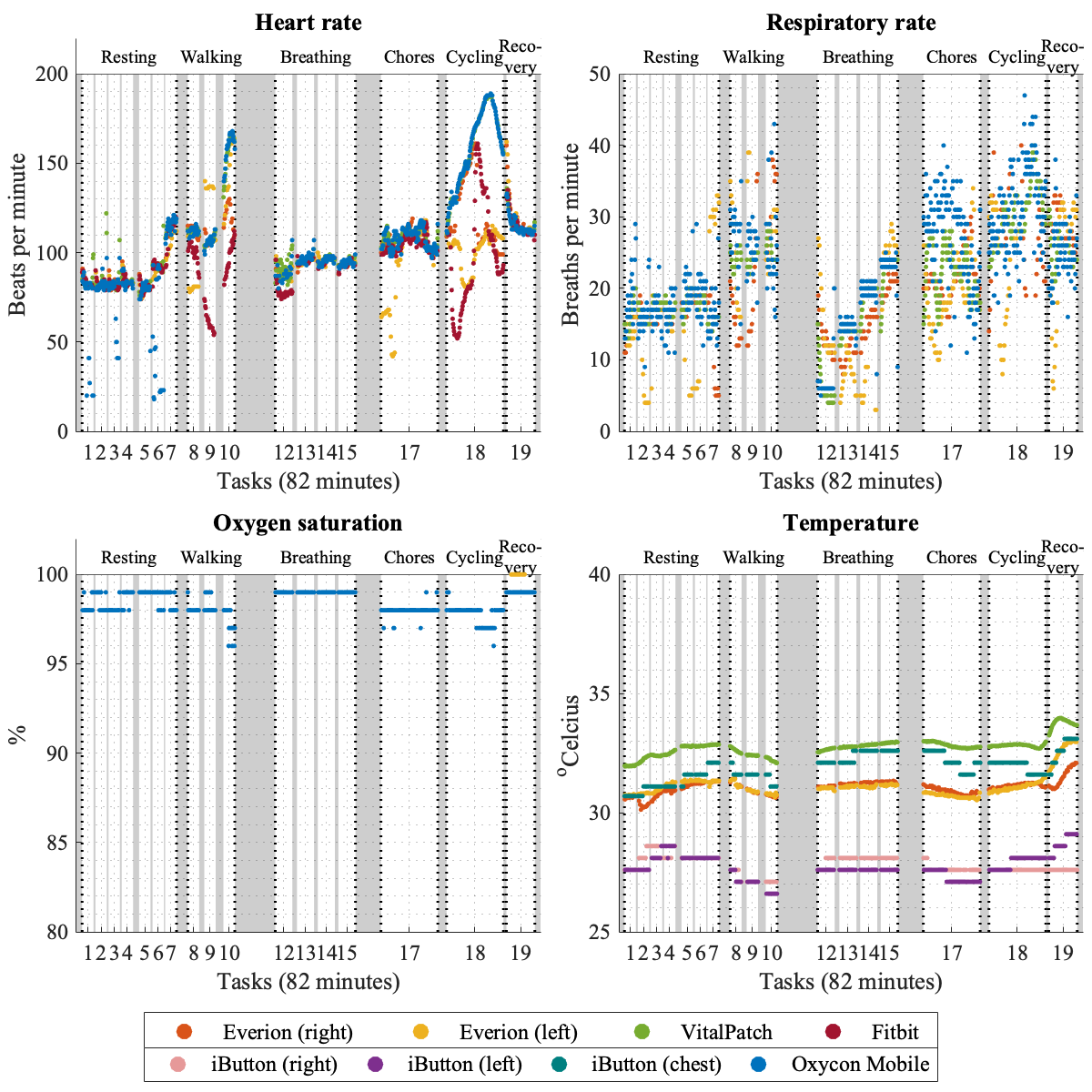

Supplement: Multimedia Appendix 2 [file formative_v6i1e30863_app2.png]
